# Editorial: Insights in genome editing tools and mechanisms: 2022

**DOI:** 10.3389/fgeed.2023.1240576

**Published:** 2023-07-11

**Authors:** Chanjuan Jiang, Qunxin She, Hailong Wang

**Affiliations:** State Key Laboratory of Microbial Technology, Institute of Microbial Technology, Shandong University, Qingdao, Shandong, China

**Keywords:** CRISPR/Cas, genome editing, peanut, *Tuta absoluta*, *Eimeria tenella*, β-thalassemia

Genome editing technologies are important tools for studying the specific functions of individual genes or modulating the expression of important genes in organisms for biological research. CRISPR (clustered regularly interspaced short palindromic repeats)-Cas (CRISPR associated) is the prokaryotic adaptive immune system that protects hosts from invading viruses and plasmids. CRISPR/Cas9 systems are the most frequently used type of genome editing tool, which is composed of the Cas9 nuclease and the guide RNA which directs Cas9 to the target DNA site by sequence complementarity. In natural systems, guide RNAs are composed of two separate RNA molecules, the CRISPR RNA (crRNA) and the trans-activating crRNA (tracrRNA), which are commonly artificially fused together to yield a single guide RNA for genome editing (Jinek et al., 2012). In addition to CRISPR/Cas9, a variety of other CRISPR-Cas systems, such as CRISPR/Cas12a (Cpf1), have been developed to overcome the difficulties of genome editing at different loci in different organisms. Recently, several new CRISPR/Cas systems have been identified and employed for genome editing, some of which are bacteriophage origin (Al-Shayeb et al., 2022). In addition, efforts have continuously been made to optimize genome editing efficiency by different CRISPR/Cas systems belonging to all six known types. Furthermore, CRISPR/Cas9 systems have been optimized for reducing their toxicity and for boosting knock-in efficiency in genome editing of primary human cells by using long single-stranded DNA homology-directed repair templates with short regions of double-stranded DNA containing Cas9 target sequences on both ends (Shy et al., 2023). This Research Topic is aimed to further explore the application of CRISPR/Cas genome editing tools in more biological systems and it includes four research articles. Three articles are under the category of *Original Research*, and one belongs to the *Brief Research Report*.

Peanut (*Arachis hypogaea* L.) seeds are the source of our daily edible oil and are rich in monounsaturated oleic acid and polyunsaturated linoleic acid. Fatty Acid Desaturase 2 (FAD2) catalyzes the conversion of oleic acid to linoleic acid. Compared with linoleic acid, oleic acid has better oxidative stability and health benefits, but increasing oleic acid content by knocking out the *FAD2* gene can lead to poor plant stress tolerance. The RY repeat element and 2S seed protein motif cis-regulatory elements in the 5′ UTR of *FAD2* genes have been suggested to have enhancer activity. Neelakandan et al. targeted these two cis-regulatory elements of the *FAD2* gene promoter by CRISPR/Cas9 to downregulate the expression levels of two homologous *FAD2* genes in seed while maintaining normal regulation in other plant tissues. Oleic acid content increased by 55%–70% in seeds of the edited RY motif and 55%–60% in seeds of the edited 2S motif compared with the wild-type seeds. The use of CRISPR/Cas9 based promoter or enhancer editing will accelerate the pace of cultivar development without introducing potentially deleterious alleles and linkage drag.

The tomato leaf miner *Tuta absoluta* (Meyrick), which is resistant to many pesticides, is one of the most destructive tomato pests in the world. To control the tomato leaf miner, intensive research on this species has been required. However, the lack of effective genome editing tools has prevented further functional genomic studies of the tomato leaf miner. Ji et al. demonstrated for the first time that CRISPR/Cas can be used for genome editing in *T. absoluta*. The CRISPR/Cas9 zygote microinjection protocol for generating heritable mutations in *T. absoluta* was developed. The injection of fertilized eggs with Cas9 protein and four sgRNAs, which targeted cinnabar exon 3, resulted in a mutagenesis rate of 31.9% for eggs reaching adulthood. The comprehensive and detailed CRISPR/Cas9 workflow for the efficient genome editing of *T. absoluta* will greatly facilitate the discovery of suitable RNAi (RNA interference) control targets and the subsequent development of novel control strategies.

*Eimeria* species can cause coccidiosis in poultry and cause significant economic losses to the poultry industry. Genome editing of *Eimeria* is of great importance for the development of vaccines and drugs. Although CRISPR/Cas9 has been used to edit the genome of *Eimeria tenella* (*E. tenella*), it requires plasmids to express CRISPR/Cas9 and would introduce transgenes, which largely limits the application in vaccine development (Hu et al., 2020; Tang et al., 2020). Cheng et al. optimized the transfection conditions for the parasite *E. tenella* and used the optimized conditions to transform the FnCas12a protein and crRNA for modification of the *EtHistone H4* gene and the *EtActin* gene. The efficiency of FnCas12a-crRNA ribonucleoprotein genome editing in *E. tenella* was 1.2%, which was lower than that (2%) obtained by integrating the Cas9 gene in the genome*.* However, the FnCas12a-crRNA method based on ribonucleoprotein transfection can avoid DNA contamination and will be advantageous for vaccines development.

β-thalassemia is a common monogenic disorder with an annual incidence of one in 100,000 worldwide. Lu et al. proposed a novel Cas9/AV6-mediated genome editing strategy for the treatment of β-thalassemia. They mimicked natural HPFH (Hereditary persistence of fetal hemoglobin) mutations −113A > G, −114C > T, −117G > A, −175T > C, −195C > G, and −198T > C by delivery of an AAV6 homology repair template, followed by immediate electroporation of Cas9-ribonucleoprotein (RNP) complexes specific to the BCL11A binding site and then successfully tested this system in both HUDEP-2 cells and HSPCs from β-thalassemia major patients. They demonstrated that introducing six specific HPFH mutations leads to significant re-activation of γ-globin and thus elevates fetal hemoglobin in both HUDEP-2 cells and HSPCs derived from β-thalassemia major patients. These findings may facilitate the clinical transformation of genome editing-based treatment of hemoglobinopathies.

CRISPR/Cas system has become the main driving force of the life science revolution in the 21st century due to its advantages in genome editing and regulation, such as low cost, stable efficiency, easy programming, and multiplex editing. However, while the CRISPR/Cas systems have great potential for genome editing, challenges remain in addressing its delivery methods, off-target activity, and inefficiencies in many organisms. With the rapid development of CRISPR/Cas tools, they will be continuously expanded in different organisms or cells to continuously meet the needs of basic biology research, gene therapy, biology breeding, and other fields.
